# Are Fried Foods Unhealthy? The Dietary Peroxidized Fatty Acid, 13-HPODE, Induces Intestinal Inflammation In Vitro and In Vivo

**DOI:** 10.3390/antiox9100926

**Published:** 2020-09-27

**Authors:** Esra’a Keewan, Chandrakala Aluganti Narasimhulu, Michael Rohr, Simran Hamid, Sampath Parthasarathy

**Affiliations:** Burnett School of Biomedical Sciences, College of Medicine, University of Central Florida, Orlando, FL 32816, USA; Esraakeewan@knights.ucf.edu (E.K.); Chandrakala.AlugantiNarasimhulu@ucf.edu (C.A.N.); mrohr1@knights.ucf.edu (M.R.); sim.hamid@knights.ucf.edu (S.H.)

**Keywords:** inflammatory bowel disease (IBD), lipid peroxide, inflammation

## Abstract

Inflammatory Bowel Disease (IBD) is a chronic inflammatory disorder characterized by progressive inflammation and the erosion of the gut mucosa. Although the exact cause of IBD is unknown, multiple factors contribute to its complex pathogenesis. Diet is one such factor and a strong correlation exists between the western-style, high fat diets (HFDs) and IBD incidence rates. In this study, we propose that the peroxidized fatty acid components of HFDs could contribute to inflammation of the gut. The inflammatory nature of peroxidized linoleic acid (13-HPODE), was confirmed in vitro by analyzing pro-inflammatory gene expression in Caco-2 cells via RT-PCR and ELISA. Additionally, peroxide induced apoptosis was tested by Annexin-V fluorescent staining, while permeability was tested by FITC-dextran flux and TEER. The 13-HPODE-induced inflammation of intestinal epithelium was evaluated in vivo by analyzing pro-inflammatory cytokines under acute and chronic conditions after feeding 13-HPODE to C57BL/6J mice. Our data show that 13-HPODE significantly induced pro-inflammatory gene expression of TNF-α and MCP-1 in vitro, most notably in differentiated Caco-2 cells. Further, acute and chronic 13-HPODE treatments of mice similarly induced pro-inflammatory cytokine expression in the epithelium of both the proximal and distal small intestines, resident immune cells in Peyer’s patches and peritoneal macrophages. The results of this study not only confirm the pro-inflammatory properties of peroxidized fats on the gut mucosa, but for the first time demonstrate their ability to differentially induce pro-inflammatory gene expression and influence permeability in the intestinal epithelium and mucosal cells. Collectively, our results suggest that the immunogenic properties of HFD’s in the gut may be partly caused by peroxide derivatives, providing potential insight into how these diets contribute to exacerbations of IBD.

## 1. Introduction

Inflammatory bowel disease (IBD) is a chronic inflammatory disorder of the gastrointestinal tract [1,2] and is categorized as either Crohn’s disease (CD) or Ulcerative Colitis (UC) depending on lesion types, pattern of progression, location and potential complications. IBD typically presents with a broad spectrum of debilitating symptoms, such as abdominal pain, cramps, diarrhea, fatigue. Patients with IBD also suffer from other complications, such as intestinal ulceration, rupture, and strictures, leading to the obstruction of the gastrointestinal tract. Moreover, patients with IBD have an increased risk of colon cancer, in addition to other systemic manifestations, which cumulatively result in serious complications that significantly impacts quality of life [3].

Although the exact etiology of IBD remains elusive, it is believed to be caused by a combination of host genetics [4], gut microbiota [5], and environmental factors [6]. In particular, diet is a major environmental factor that may contribute to the development of IBD. For example, it has been noted that IBD incidence rates are greatest in western nations and are on the rise in developing nations, such as India, as industrialization is rapidly westernizing dietary trends [7,8]. Western-style diets are high in fat and carbohydrates, while being low in fiber. Studies have consistently shown that this dietary composition not only can induce intestinal inflammation on its own [9,10], but also aggravate pre-existing inflammation and enhance intestinal permeability in mouse models and even humans [7,11,12]. In fact, it is believed that the high fat component is a major immunogenic factor, as patients with IBD are more likely to experience flare-ups when consuming fatty foods and is a major reason for why IBD-specific diets either significantly reduce or eliminate fats [13]. However, although the immunogenicity of fats in the intestinal system are well documented [7], little is known about specific causative agents and how they contribute to the observed inflammatory response. A strong candidate to explain this process comes in the form of dietary peroxidized lipids (POLs). 

POLs are by-products of exogenous or endogenous oxidation of polyunsaturated fatty acids (PUFAs) double bonds, which are abundant in western-style diets. Both natural processes like autoxidation and artificial processes, such as deep-frying and long-term exposure to air result in the oxidation of PUFAs to POLs. [14]. Linoleic acid is an omega-6 fatty acid and is the most abundant PUFA found in plants and mammals [15] and its oxidation yields 13-hydroperoxy octadecadienoic acid (13-HPODE) as a major product. Previous studies from our laboratory have shown that 13-HPODE decomposes to toxic aldehydes and carboxylic acids under physiologic conditions [16]. In addition, studies by us and others have demonstrated potent pro-inflammatory properties of 13-HPODE and other POLs [17,18,19]. Importantly, as the small intestines serve as a first-line barrier to dietary products and toxins, the gut mucosa experiences the largest concentrations of POLs which we hypothesize may underly the intestinal inflammation observed with the high fat diets (HFDs). Therefore, in this study, we tested the immunogenic properties of the most common dietary POL, 13-HPODE, on intestinal epithelium both in vitro and in vivo.

## 2. Materials and Methods

### 2.1. Materials

Linoleic acid, soybean lipoxygenase type V and other chemicals were purchased from Sigma (St. Louis, MO). Leucomethylene blue (LMB) was obtained from Alfa Aesar (Ward Hill, MA, USA). Trizol^TM^ reagent and primers was purchased from Invitrogen (Carlsbad, CA, USA). All ELISA kits were purchased from R&D Systems (Minneapolis, MN, USA).

### 2.2. Cell Culture

Caco-2 and THP-1 cells were purchased from American Type Culture Collection (ATCC) (Rockville, MD, USA). Cells were cultured in Dulbecco’s modified eagle medium (DMEM) supplemented with 15% fetal bovine serum (FBS), 2 mM L-glutamine, 1X penicillin-streptomycin, and 1X Non-Essential Amino Acids. After attaining confluence, cells were cultured in the same medium supplemented with 7.5% FBS while keeping the other constituents the same. Confluent cells were trypsinized using 0.25% Trypsin-EDTA solution. Caco-2 cells were seeded in 6- and 12-well plates depending on the experiment. Experiments were carried out on days 3-5 (undifferentiated cells) and 14-21 (fully differentiated cells) after each passage. In order to ensure confluence on days 3-5, cells were seeded at a higher density.

### 2.3. Preparation of HPODE

200 µM 13-hydroperoxy octadecadienoic acid (13-HPODE) was prepared, as described previously [20]. Lipid peroxide generated in the reaction system was analyzed by LMB assay [16] and the amount of peroxide generated was quantitated. The formation of 13-HPODE was confirmed by measuring conjugated diene reading at 234 nm by a Jenway (Coleparmer, UK) spectrophotometer, and peroxide was confirmed by LMB assay using Bio-Rad plate reader (Bio-Rad Benchmark Plus). Freshly prepared 13-HPODE was filter-sterilized and used within two hours for experimentation to minimize the risk of contamination as well as spontaneous peroxide decomposition.

For animal studies, 25g of 13-HPODE were prepared and confirmed similarly. The oxidized fatty acid was extracted with Butylated hydroxy toluene (BHT) free ether, dried under the stream of nitrogen, and stored at −20 °C until use.

### 2.4. Treatment of Cells with 13-HPODE

Caco-2 cells (undifferentiated and differentiated) were seeded at an initial density of 2 × 10^4^ cells per well. Experiments were carried out on days 3–5 days for undifferentiated Caco-2 cells, and 14–21 days for fully differentiated Caco-2 cells. Cell differentiation was confirmed by measuring intestinal alkaline phosphatase (ALPI) expression over time. Cells were starved in serum-free medium for 3 h prior to the treatments. Cells were treated with increasing concentrations of 5–100 µM 13-HPODE for 24 h, to which 50 and 100 µM 13-HPODE were chosen for further experiments. After 24 h incubation, cells were harvested into Trizol for RNA isolation and the medium was collected, stored at −80 °C, and used for ELISA.

### 2.5. Chemotaxis of THP-1 Cells and Conditioned Media

To evaluate whether 13-HPODE itself or the cytokine milieu produced by Caco-2 cells were chemoattractants, chemotaxis of THP-1 monocytes was assayed. Prior to chemotaxis, THP-1 cells were starved for 4 h and labeled with 1,1′-Dioctadecyl-3,3,3′,3′-Tetramethylindocarbocyanine Perchlorate (DiIC_18_) fluorescent membrane dye for 15 min. Afterwards, THP-1 cells were added to the top chamber of a transwell filter system (0.8 µm pore size) in serum free media; the bottom chamber was filled with either 13-HPODE containing media or media conditioned from Caco-2 cells pre-treated with 13-HPODE for 24 hrs. THP-1 cells were allowed to migrate for 12 h and adherent cells were disaggregated from the filter by incubation in 0.5% Ethylenediaminetetraacetic acid (EDTA) in Phosphate buffer saline (PBS). The number of cells in the bottom chamber were quantitated using a CytoFLEX 2 flow cytometer (Beckman Coulter, Brea, CA, USA). Data were reported as fold change in cell counts relative to control.

Conditioned media (CM) were prepared from Caco-2 cells by harvesting media after the treatment time-point, followed by centrifugation (3000 rpm for 10 min) and syringe filtration to remove debris. CM was used within 8 h after collection and never frozen to preserve the cytokine milieu.

### 2.6. Annexin Staining

To evaluate 13-HPODE-induced apoptosis in undifferentiated and differentiated Caco-2 cells following treatment of 13-HPODE, cells were stained with Annexin V-FITC and propidium iodide (PI) (Sigma, St. Louis, MO, USA) according to the supplier’s protocol. Briefly, cells were washed twice with PBS and resuspended in binding buffer. They were then incubated with Annexin V-FITC (5 μL) and PI (10 μL) in the dark for 15 min at room temperature. Cells were then washed with binding buffer and resuspended in a fresh binding buffer. The cell suspension was spotted on a glass slide covered by a coverslip and visualized using a Leica fluorescence microscope.

### 2.7. Transepithelial Electrical Resistance (TEER) Measurements

Caco-2 cells were seeded on a 0.4 μm polyester trans well filter support (Corning-Sigma, Burlington, MA, USA). Caco-2 cells were grown for 14 days to ensure complete monolayer formation with confirmation determined by a resistance between 400–600 Ω∙cm^2^ using a Millicell ERS-2 Volt/ohm Meter (Millipore, Burlington, MA, USA) with chopstick electrodes. For experimentation, Caco-2 cells were starved in serum-free medium for 3 h followed by the replenishment of media and incubation of compounds. TEER was measured both before and after the treatment duration with measurements being performed at room temperature to minimize the effect of heat on resistance. All TEER values were reported as the average of three separate measurements at each time point minus the TEER of a blank filter with no cells multiplied by the area (in cm2) of the filter. The final value was reported as the percent TEER relative to time = 0 h.

### 2.8. Fluorescein Isothiocyanate (FITC)-Dextran Flux

Permeation to uncharged molecules was assessed via unidirectional 4 kDa FITC-dextran (Sigma, Burlington, MA, USA) flux. After incubation of filter-grown Caco-2 monolayers with 13-HPODE, monolayers were washed with PBS and supplied with fresh phenol red free-serum free medium. Thereafter, 1 μg/mL FITC-dextran was added to the top chamber in which 100 μL aliquots were removed from the bottom well and added to a black-bottom 96-well plate (Corning, New York, NY, USA) in triplicates at indicated time points. An equal amount of medium was returned to the bottom wells to preserve volume. Fluorescence was measured in a PerkinElmer Envision 2104 Multilabel Plate Reader at an excitation wavelength of 490 nm and emission wavelength of 520 nm. For consistency, fluorescent flux values obtained 30 min after addition of dextran to top well were used.

### 2.9. Animals

Twelve and seventeen, 4-week-old male C57BL/6J mice weighing 18–20 g were obtained from Jackson Laboratory (Bar Harbor, ME, USA) acclimatized and at the age of 10 ± 2 weeks, used for acute and chronic inflammation studies, respectively. All procedures were performed according to the protocol approved by The Institutional Animal Care and Use Committee of the University of Central Florida and all methods were performed in accordance with the relevant guidelines and regulations (16-18/7.13.2016).

### 2.10. Acute Inflammation

For acute studies, mice were divided into 2 groups (*n* = 6 pergroup), including group 1 as control and group 2 as HPODE. After overnight fasting mice were fed with 250 mg 13-HPODE in 400 µL of PBS through oral gavage and left for 4 h and euthanized.

### 2.11. Chronic Inflammation

For chronic studies, four animals were sacrificed to obtain baseline parameters. The remaining animals were divided into two groups: group 1 (*n* = 6) was fed with normal diet, group 2 (*n* = 7) was fed normal diet supplemented with peroxidized fat (NCP: HPODE), for one month. After one-month, animals were sacrificed. The animals were regularly monitored, and weekly measurements of body weights were recorded until sacrifice at day 30.

### 2.12. Diet

The normal Purina diet (TD.150278), was purchased from Harlan Teklad (Madison, WI, USA). Normal diet (NC) was supplemented with peroxidized fat (NCP (40mg/day/mouse-prepared in-house)) and allowed for drying. Diets were stored at 4 °C to avoid oxidation. Diet was weighed and supplied on a daily basis. Average food consumption was recorded on a daily basis until sacrifice at day 30.

### 2.13. Isolation of Mouse Peritoneal Macrophages

Mouse peritoneal macrophages were isolated as described previously by us [21,22,23]. Briefly, macrophages from the peritoneal cavity of all groups of animals were isolated by peritoneal lavage using 3 mL of cold saline and centrifugation at 1200 rpm for 5 min. Cells were utilized for RNA isolation.

### 2.14. Collection of Plasma and Organs

For acute studies, blood samples were collected after 4h of treatment. At the end of the experiments, mice were fasted overnight for chronic studies and anesthetized with 1–2% isoflurane. Blood samples were collected into EDTA tubes by heart puncture. Plasma was separated as described previously [24] and stored at –80 °C and mice were euthanized using carbon dioxide asphyxiation. Intestines were harvested, cleaned, proximal and distal intestines were identified and flushed with ice cold PBS followed by isolation of intestinal epithelium (IE) and Peyer’s patch (PP) tissue. Tissue samples were washed with PBS and were immediately snap frozen for RNA extraction.

### 2.15. Plasma Lipid Analysis

Plasma lipid profiles of total cholesterol (TC), triglyceride (TRG), HDL cholesterol (HDLc), and LDL cholesterol (LDLc) were determined using a Cholestech L*D*X analyzer (Cholestech Corp, Hayward, CA, USA).

### 2.16. cDNA Synthesis and RT-PCR Reaction

Total RNA from undifferentiated and differentiated Caco-2 cells, intestinal tissues (IE and PP) was isolated by using TrizolTM reagent. Then, 1 µg of RNA was reverse transcribed into cDNA using the SuperscriptTM III First Strand Synthesis System (Invitrogen, Carlsbad, CA, USA). Then, 50 ng cDNA was used to perform Quantitative real-time PCR by iQTM5 iCycler and CFX96 Multicolor Real-Time PCR Detection System (Bio-Rad, Hercules, CA) with SYBR Green (Invitrogen, Carlsbad, CA, USA). Mouse and human oligonucleotide primers for RT-PCR were purchased from Invitrogen (Carlsbad, CA, USA). Real time polymerase chain reaction (PCR) was carried out with ALPI, Claudin-2, interleukin (IL)-4, IL-6, Monocyte chemoattractant protein-1 (MCP-1), Occludin and Tumor necrosis factor alpha (TNF-α) with human and mouse-specific primers (Supplementary Table S1), resulting in a 200 bp fragment. As a reference gene, we used Glyceraldehyde-3-Phosphate Dehydrogenase (GAPDH) primers, resulting in a 200-bp fragment. PCR was performed with an initial step of denaturation at 50 °C for 2 min and 95 °C for 10 min, followed by 40 cycles of 95 °C for 20 s and 60 °C for 20 s. Melt curves were established for the reactions. Normalized fold expression was calculated by using the 2-∆∆Ct method.

### 2.17. Enzyme-Linked Immunosorbent Assay (ELISA)

Medium was collected from undifferentiated as well as fully differentiated Caco-2 cells under different experimental conditions and used. Fifty microliters of samples were analyzed using sandwich enzyme-linked immunosorbent assay (ELISA) kits (R&D systems, Minneapolis, MN, USA) TNF-α, MCP-1, and IL-4 following suppliers’ protocol.

### 2.18. Global Cytokine Array

Plasma samples were analyzed by the global cytokine array by Ray Biotech Inc. (Norcross, GA, USA) using RayBio^®®^Mouse G Series Inflammation Array 1.

### 2.19. Statistics

Values are presented as mean ± SEM, and statistical analysis were performed by using Student t-test at significance of *p* < 0.05. All data were analyzed using GraphPad Prism 6 software (GraphPad Software, San Diego, CA, USA).

## 3. Results

### 3.1. 13-HPODE-Induced Pro-inflammatory Cytokines in Undifferentiated as Well as Differentiated Caco-2 Cells

**Figure 1:**
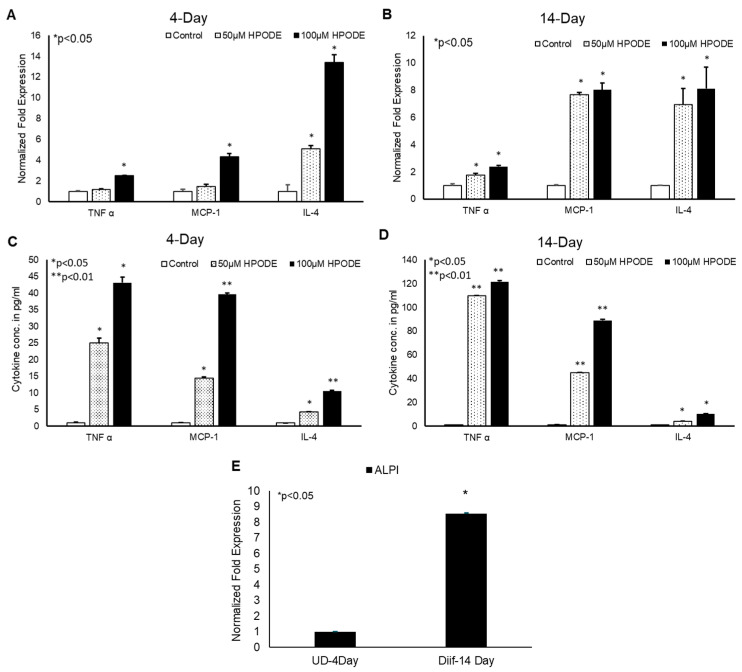
13-HPODE induces pro-inflammatory gene expression in undifferentiated and differentiated Caco-2 cells. (**A**,**B**) 4-day (undifferentiated) and 14-day (differentiated) Caco-2 cells were incubated with increasing concentrations (50 and 100 µM) of 13-HPODE (HPODE) for 24 h. RNA was isolated and qPCR analysis was performed assessing for pro-inflammatory TNF-α, MCP-1, and IL-4 gene expressions using appropriate primers. (**C**,**D**) Secretion of pro-inflammatory cytokine levels was measured by ELISA. (**E**). Caco-2 cell differentiation was confirmed by ALPI expression. Results are represented as mean ± SEM and significance was considered as * *p* < 0.05, ** *p* < 0.01. TNF-α, Tumor necrosis factor alpha; MCP-1, Monocyte chemoattractant protein-1; IL-4, Interleukin-4; ALPI, Intestinal alkaline phosphatase.

To determine the immunogenic effect of 13-HPODE on intestinal epithelium in vitro, undifferentiated (4-day) and differentiated (14-day) Caco-2 cells were treated with 50 and 100 µM 13-HPODE for 24 h to which the expression levels of pro-inflammatory cytokines were then measured. We found that 13-HPODE directly induced TNF-α, MCP-1, and IL-4 expression in both 13-HPODE treated undifferentiated (Figure 1A) and differentiated (Figure 1B) Caco-2 cells. Further analysis of media cytokine levels via ELISA were consistent with expression patterns (Figure 1C,D). Differentiation was confirmed by ALPI expression (Figure 1E). Moreover, we found that 13-HPODE and media conditioned from 13-HPODE-treated Caco-2 cells enhanced THP-1 cell chemotaxis (Supplemental Figure S1A,B) confirming that POLs not only induce inflammation, but also may contribute to attract the immune cells to the affected site.

### 3.2. Annexin V Staining of 4-Day and 14-Day Cells Incubated with 13-HPODE

**Figure 2:**
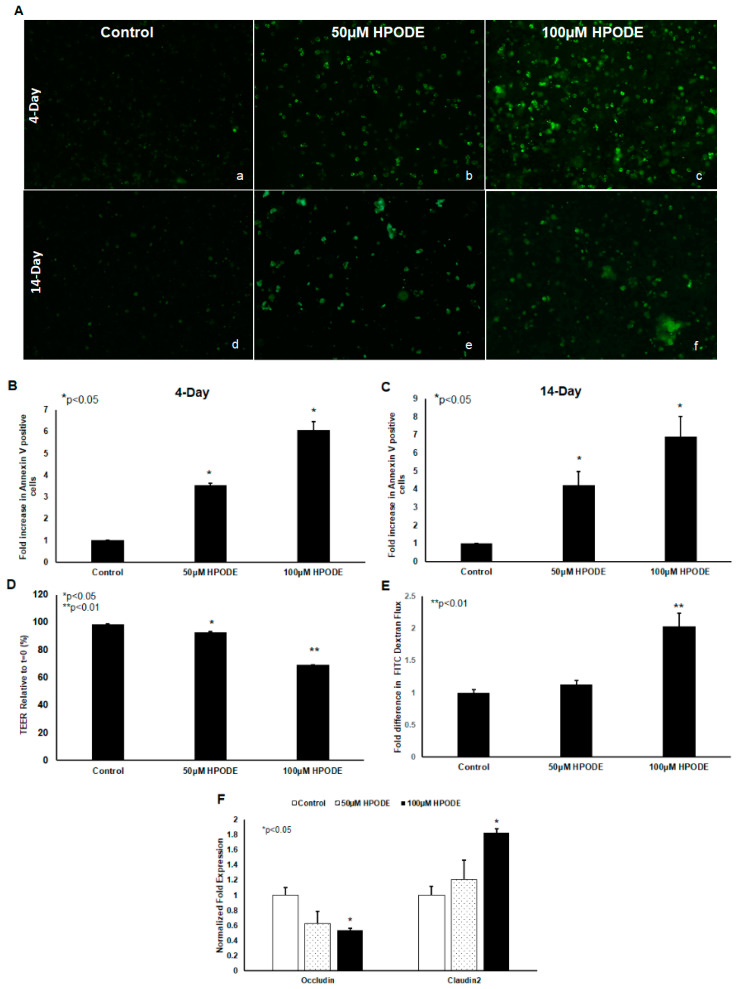
13-HPODE enhances Caco-2 cell apoptosis (undifferentiated and differentiated cells) and permeability (differentiated cells). Undifferentiated and differentiated Caco-2 cells were incubated with 13-HPODE (HPODE) for 24 h at 37 °C. Annexin V-FITC staining was performed to evaluate the apoptosis and images were recorded, (**A**) (**a**–**f**). Fluorescence quantification was analyzed by ImageJ (**B**,**C**). Caco-2 permeability as assessed by TEER measurement (**D**) and FITC dextran flux (**E**). qPCR analysis for tight junction genes Occludin and Claudin-2 in Caco-2 cells treated with 13-HPODE (**F**). Results are represented as mean ± SEM and significance considered as * *p* < 0.05, ** *p* < 0.01. FITC, Fluorescein isothiocyanate; TEER, Transepithelial electrical resistance.

POLS are known to cause cytotoxic oxidative stress and thus we tested the impact of 13-HPODE on Caco-2 cell viability. We found that 13-HPODE significantly induced apoptosis in undifferentiated and differentiated Caco-2 cells, as assessed by annexin V staining (Figure 2A–C). In addition, because enterocyte oxidative stress and apoptosis are both intrinsically linked to gut barrier dysfunction [7], we next assessed the impact of 13-HPODE on Caco-2 monolayer permeability. As expected, 13-HPODE enhanced permeability, which was reflected by a significant reduction in TEER (Figure 2E) an elevation in FITC-dextran flux (Figure 2F), and reduced barrier-promoting Occludin and increased pore-forming Claudin-2 expression (Figure 2G).

### 3.3. In Vivo Studies

#### 3.3.1. Metabolic Characteristics of Mice

**Figure 3:**
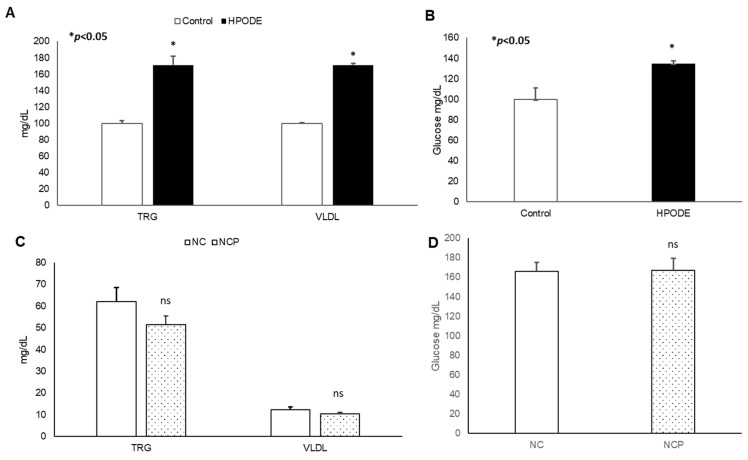
Plasma lipid and glucose levels of mice. Plasma lipid and glucose levels in 13-HPODE diet-fed animals in (**A**,**B**) acute and (**C**,**D**) chronic conditions Values are represented as mean ± SEM. Significance was considered as * *p* < 0.05, ns: not-significant. NC, Normal chow; NCP, Normal chow supplemented peroxidized fat; TRG, Triglycerides; VLDL, Very low-density lipoprotein.

No significant changes in body weight was observed in either acute inflammation or chronic inflammation studies (data not shown). Similarly, in chronic studies, no significant difference was observed in average diet consumption between groups (data not shown). In acute studies, a significant increase in total triglyceride (TRG), very low-density lipoprotein (VLDL), and glucose was observed during the 4 h treatment (Figure 3A,B). Noticeably, no significant changes were observed in the metabolic serum profiles of NC and NCP diet-fed mice (Figure 3C,D).

#### 3.3.2. Gene Analysis of Mice Proximal and Distal IEs and PPs

**Figure 4:**
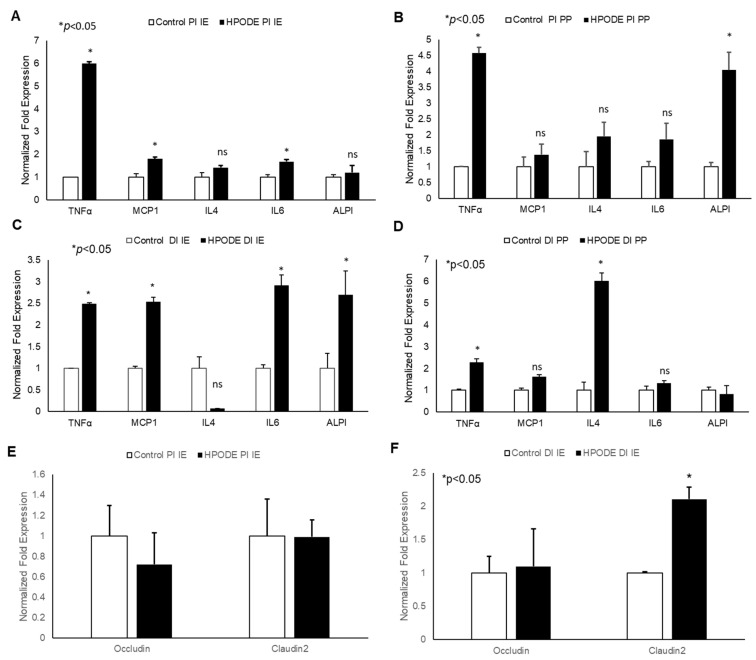
Acute effect of 13-HPODE on murine proximal and distal IE and PP. In acute studies, C57BL/6J mice received either PBS (control group) or 13-HPODE (HPODE group) via oral gavage and were euthanized after 4 h. Following sacrifice, the small intestine was collected, proximal (PI) and distal (DI) portions were separated, from which intestinal epithelium (IE) and Peyer’s patches (PP) were collected. (**A**,**B**) PI-, (**C**,**D**) DI-Gene expression of pro-inflammatory and (**E**,**F**) permeability markers in PI/DI IE and PP were assessed by qPCR. Results are represented as mean ± SEM and significance was considered as * *p* < 0.05, ns: not-significant. TNF-α, Tumor necrosis factor alpha; MCP-1, Monocyte chemoattractant protein-1; IL, Interleukin; ALPI, Intestinal alkaline phosphatase; PI, Proximal intestine; DI, Distal intestine; IE, Intestinal epithelial tissue; PP, Peyer’s patches.

In acute studies, a significant increase in TNF-α gene expression was observed in proximal (PI) and distal (DI) intestinal epithelial tissues, as well as in Peyer’s patches (PP) in 13-HPODE treated mice (Figure 4A–D). Similarly, a significant induction in MCP-1 and IL-6 was observed in PI and DI epithelial tissue, whereas a non-significant increase was observed in PPs. The 13-HPODE treatment also resulted in reduced IL-4 expression in DI and increased expression in DI PP; no changes were seen in PI tissue (Figure 4A–D). Similarly, a significant increase in ALPI gene expression was observed in distal IE, as compared to the control.

No significant changes were observed in permeability (as assessed by Occludin and Claudin-2 expression) in PI epithelium, but changes were observed in DI epithelium as early 4 h, suggesting that POLs are able to influence the gut barriers even at short exposures (Figure 4E,F).

**Figure 5:**
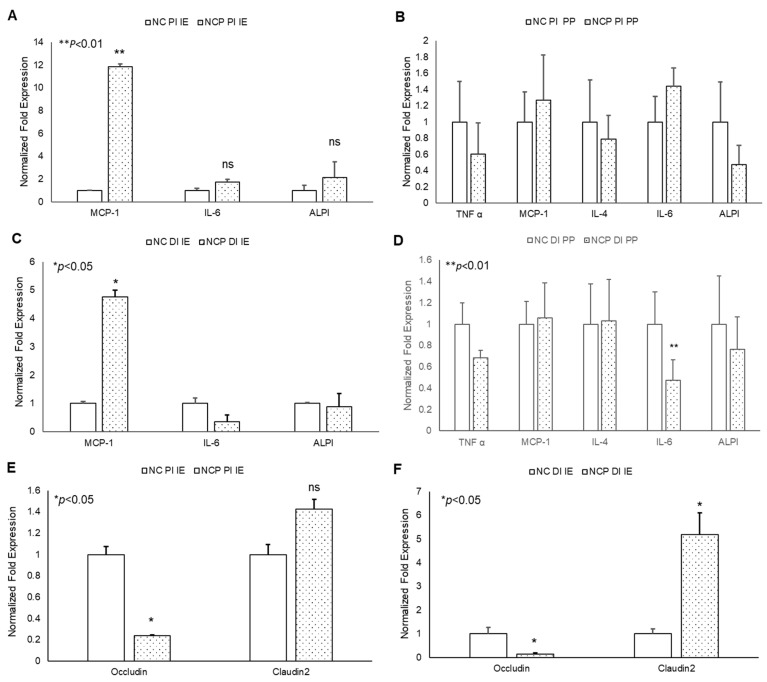
Chronic effects of 13-HPODE on murine proximal and distal IE and PP. For chronic studies, mice were fed with normal diet (NC) or a diet containing 4 mg/kg/mouse 13-HPODE (NCP) for one month followed by overnight fasting and euthanasia. Following sacrifice, the small intestine was collected, proximal (PI) and distal (DI) portions were separated, the intestinal epithelium and PP’s were collected. (**A**,**B**) PI- (**C**,**D**) DI- Gene expression of pro-inflammatory, and (**E**,**F**) permeability markers of PI/DI were assessed by qPCR. Results are represented as mean ± SEM and significance was considered as * *p* < 0.05, ** *p* < 0.01 ns: not-significant. TNF-α, Tumor necrosis factor alpha; MCP-1, Monocyte chemoattractant protein-1; IL, Interleukin; ALPI, Intestinal alkaline phosphatase; PI, Proximal intestine; DI, Distal intestine; IE, intestinal epithelial tissue; PP, Peyer’s patches; NC, Normal chow; NCP, Normal chow supplemented with peroxidized fat.

The chronic treatment of mice with peroxide-spiked diet revealed a significant increase in MCP-1 expression in both PI and DI epithelium, but not PP tissue, compared to control (Figure 5A–D). No significant changes were observed in other inflammatory cytokines. In addition, a significant decrease in Occludin and increase in Claudin-2 gene expression was observed in 13-HPODE treated PI and DI IE epithelium, suggesting greater intestinal permeability persisting under chronic conditions (Figure 5E,F). These data are consistent with our in vitro permeability studies and further confirm the potent impact of dietary POLs on mucosal barrier dysfunction.

#### 3.3.3. Gene Expression in Mouse Peritoneal Macrophages

**Figure 6:**
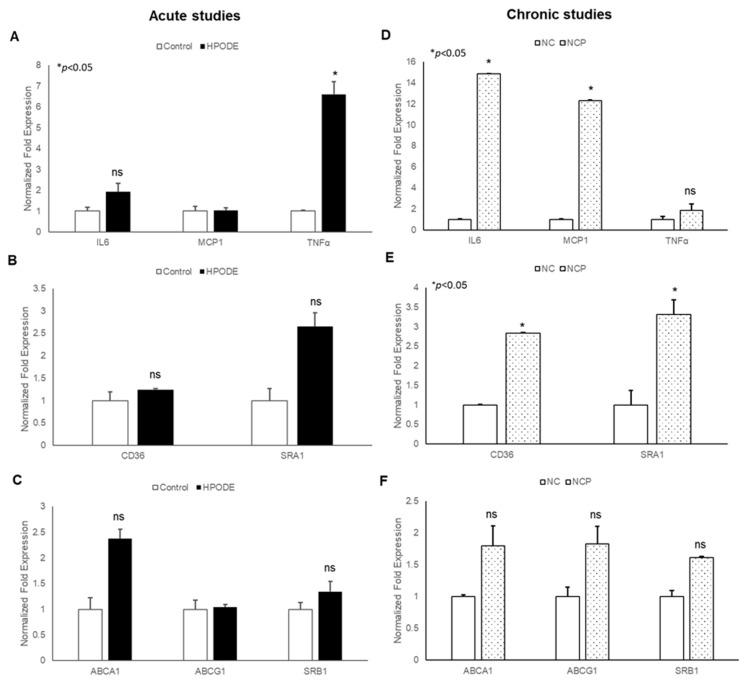
Gene expression analysis of peritoneal macrophages isolated from peroxidized linoleic acid (13-HPODE) fed mice in acute and chronic studies. In acute studies, C57BL/6J mice received either PBS (control group) or 13-HPODE (HPODE group) via oral gavage and were euthanized after 4 h. For chronic studies, mice were fed with normal diet (NC) or a diet containing 4 mg/kg/mouse 13-HPODE (NCP) for one month followed by overnight fasting and euthanasia. Following sacrifice, peritoneal macrophages were isolated and gene expression was analyzed by qPCR. Bar diagrams represent pro-inflammatory gene expressions in (**A**) acute and (**B**) chronic Scavenger receptors; (**C**) acute and (**D**) chronic studies; RCT genes; (**E**) acute and (**F**) chronic studies. Values are represented as mean ± SEM and significance was considered as * *p* < 0.05, ns: not-significant. TNF-α, Tumor necrosis factor alpha; MCP-1, Monocyte chemoattractant protein-1; IL, Interleukin; CD36, cluster of differentiation 36; ABCA1, ATP Binding Cassette Subfamily A Member 1; ABCG1, ATP Binding Cassette Subfamily G Member 1; SRA1, Scavenger receptor A1; SRB1, Scavenger receptor B1; NC, Normal chow; NCP, Normal chow supplemented with peroxidized fat.

Our findings that 13-HPODE induces THP-1 chemotaxis prompted us to evaluate the effects of 13-HPODE murine peritoneal macrophages (PMs). We subsequently assessed gene expression of pro-inflammatory cytokines and reverse cholesterol transport (RCT) proteins in POL-treated mice in both acute and chronic settings. In acute studies, we observed a non-significant increase in pro-inflammatory cytokine TNF-α, cholesterol transport gene (ABCA1: ATP Binding Cassette Subfamily A Member 1), and scavenger receptor A1 (SRA1) as compared to control PMs. However, no change was observed in other inflammation, associated as well with RCT (Figure 6A–C).

The results of chronic studies showed that PMs isolated from peroxidized fat-treated animals had significantly increased gene expression of inflammatory cytokines (IL-6 and MCP-1) and cholesterol uptake proteins (CD36: cluster of differentiation 36; SR-A1) with no changes observed in RCT genes (ABCA1, ABCG1: ATP Binding Cassette Subfamily G Member 1; SRB1: Scavenger receptor B1). Enhanced cholesterol uptake proteins with no changes in RCT suggest further enhancement of cholesterol uptake and increased inflammation. Collectively, these results confirm that the immunogenicity of 13-HPODE extends beyond the intestinal epithelium.

#### 3.3.4. Cytokine Array

Cytokine array data revealed that treatment of mice with 13-HPODE induced an increase in serum pro-inflammatory cytokines, including Granulocyte colony stimulating factor (GCSF), Macrophage colony stimulating factor (MCSF), IL-1beta, Regulated upon Activation, Normal T Cell Expressed and Presumably Secreted (RANTES), soluble tumor necrosis factor receptor 1 (sTNFRI), and sTNFRII (Table 1) in 13-HPODE treated mice, as compared to the controls. The cytokine milieu confirms active inflammation caused by feeding with POLs and validates in vitro findings.

## 4. Discussion

In contrast to fatty acids, which are absorbed in the proximal gut, peroxidized lipids (POLs) resemble lithocholic acid and are absorbed proximally, distally, and even in the large intestines [25]. Importantly, many POLs have been reported to be pro-inflammatory in literature [26,27,28] and thus it is possible that the oxidative capability of POLs may play a role in gut inflammation. Thus, in the present study, we tested the hypothesis that dietary POLs induce intestinal and gut mucosal inflammation in vitro and in vivo. We found that treatment of undifferentiated and fully differentiated Caco-2 intestinal cells with 13-HPODE potently induced pro-inflammatory gene expression and cellular apoptosis in a dose-dependent manner. Furthermore, we observed that the acute and chronic feeding of mice with a 13-HPODE spiked diet resulted in significant epithelial inflammation and the activation of peritoneal macrophages that localized to the distal intestines, similar to what is observed with CD. Lastly, cytokine array revealed that, in addition to MCP-1, IL-4, and TNF-α, other cytokines were upregulated, such as BLC, GCSF, and TIMP-1, in mouse serum. Collectively, we believe that the results from this study may shed light on the inherent immunogenicity of HFDs and reveal novel causative factors that may play a major role in the etiology of IBD.

Existing studies suggest that both resident immune cells and intestinal epithelial (IE) cells play an essential role in the development of mucosal inflammation as a result of chronic HFD-consumption and even IBD [29]. It is agreed that the IE plays an important role in coordinating and maintaining inflammatory responses [29]. Therefore, we tested the effects of 13-HPODE on Caco-2 cells and mouse intestines. Of note, 13-HPODE induced the expression and secretion of the Th1 cytokines IL-6, MCP-1, and TNF-α both in both Caco-2 cells and mouse IE and PP tissues. Of note, we found that, of the cytokines tested, 13-HPODE potently induces both Th1 cytokines, including TNF-α, MCP-1 and IL-6. These cytokines play major roles in the development and progression of inflammatory gut diseases and have been intrinsically linked to HFD-induced mucosal inflammation. For example, elevated MCP-1 may suggest that dietary POLs promote monocyte recruitment and their differentiation into macrophages in the presence of TNF-α and IL-6. In the presence of other cytokines, these macrophages may, in turn, produce more TNF-α, resulting in sustained inflammation. Additionally, we similarly found significantly elevated expression levels of the Th2 cytokine, IL-4. As with Th1-type cytokines, IL-4 is also known to be highly immunogenic and is commonly elevated in colitis. For example, Van K.C. et al. [30] showed that IL-4 has the ability to act as a pro-inflammatory cytokine in the mucosa of the colon and could lead to colitis. In addition, Specht et al. [31] demonstrated that the spontaneous development of colitis was of a significantly lower degree in IL-4/IL-10 double deficient mice, as compared to IL-10 deficient mice, which developed severe colitis. Thus, peroxidized lipids may induce colitis through IL-4, which in the case of humans, would correspond to a UC-like Th2-like response. However, IL-4 levels were not well detectable in 13-HPODE treated medium by ELISA. This might be due to the time-lapse in the expression of gene and the secretion of the protein. IL-4 might be in the transcriptional phase for the duration of the exposure of 13-HPODE and might not be secreted into medium for the duration of treatment. Another possibility might be due to the short half-life of IL-4. For example, previous studies have shown that un-complexed IL-4 has a very short half-life (<15 min) [32,33]. Other studies have shown that natural sIL-4Rα and rIL-4 complexes rapidly dissociate and the half-life of IL-4/sIL-4Rα formed from its dissociation is ~2 h [33]. Further investigations are needed to determine the discrepancies in IL-4 gene and cytokines expression levels.

Interestingly, we observed that 13-HPODE-induced intestinal inflammation tended to localize to distal sites, especially in the acute time-points, which is a feature observed in terminal ileitis [34]. Because the distal IE is enriched in immune cells, taking form as lymphoid follicles or Peyer’s patches (PPs), we hypothesized that, in addition to the IE, resident immune cells could also be affected by POL feeding. We found that feeding mice with 13-HPODE spiked diets resulted in cytokine profiles within PPs that were similar to those in the IE. In addition, we found increased expression of pro-inflammatory genes in peritoneal macrophages isolated from 13-HPODE treated mice compared to controls. Moreover, 13-HPODE enhanced THP-1 monocyte chemoattraction, reiterating results by Rolin et al., 2013 [35], showing similar activity in natural killer cells. These results indicate that, in addition to the IE, dietary POLs may also drive inflammation through directly attracting resident immune cells and stimulating their activity. Indeed, this was shown by Fujiyama et al., 2007 [36], who found the enhanced homing of leukocyte to the gut mucosa upon feeding mice with lard. We believe that dietary POLs may be responsible for these findings. Although unoxidized dietary fats are known to have similar effects [9,10], this is the first study to show that POLs have similar immunogenic functions within the IE and thus may be as important as regular fats in promoting intestinal immune responses.

Another means by which POLs may negatively impact IE homeostasis and induce inflammation is through the progressive erosion of the epithelial barrier. Impaired barrier integrity results in the permeation of luminal antigens, which stimulate potent inflammatory responses by resident immune cells and has been intrinsically linked with HFD consumption [7] and is thought to be the precursor to the observed intestinal inflammation and low-grade systemic inflammation that is typically found in obese individuals [12] and even in patients with IBD [37]. We hypothesized that POLs may contribute to enhanced intestinal permeability through direct cytotoxicity. Previous reports from our laboratory have shown that 13-HPODE spontaneously decomposes to cytotoxic aldehydes and carboxylic acids under physiologic conditions [16]. Indeed, the treatment of Caco-2 cells enhanced apoptosis and elevated both transcellular and paracellular monolayer permeability. In addition, we also found that 13-HPODE treatment induced “claudin-switching,” which involves the downregulation of barrier-forming tight junction proteins (TJPs), such as Claudin-1 and Occludin, and the subsequent upregulation of pore-forming Claudin-2. This phenomenon is often associated with inflammatory diseases, including IBD, and is thought to occur through the cytokine-mediated dysregulation of TJP expression [38]. While the specific mechanism(s) underlying our observations are not yet fully understood, the implications of our findings are intriguing and therapeutically relevant. First, HFDs are rich in PUFAs and exogenous and endogenous oxidation by food preparation or the gastric chemical environment, respectively [39,40,41], result in the delivery of POLs to the small intestines in high quantities. Second, earlier studies from our laboratory show that oxidized fatty acids are efficiently taken up by intestinal cells in a dose-dependent manner and are governed by the expression of brush border enzymes [26,42]. Combined with the fact that peroxides may induce cellular cytotoxicity through interaction with the lipid bilayer, it is possible that POLs damage the IE within the enterocytes and the extracellular space (intestinal lumen), both potently inducing IE inflammatory responses.

Previous studies from our laboratory have shown that 13-HPODE spontaneously decomposes to cytotoxic aldehydes and beneficial carboxylic acids under physiologic conditions [16]. However, more studies are needed to investigate this notion and we hope that the results from this investigation open avenues in this regard.

Our previous studies with endothelial cells suggested that 13-HPODE may also act to suppress oxidative stress by promoting the expression of antioxidant genes [20,43]. While such beneficial responses could be cell-specific or time/concentration dependent, studies have also found that 13-HPODE induces the expression of VCAM-1 and other pro-inflammatory genes in endothelial cells [17]. Our in vivo studies suggest that the pro-inflammatory actions could be sustained and could also contribute to the systemic pathophysiology. In fact, our previous studies on cholesterol-fed mice showed that the presence of 13-HPODE increased cholesterol absorption and the atherogenicity of dietary cholesterol. What was striking in those studies was that, even when plasma cholesterol levels were adjusted to be similar, 13-HPODE-treated animals showed increased atherosclerosis, a disease that is closely associated with inflammation [26].

## 5. Conclusions

**Figure 7:**
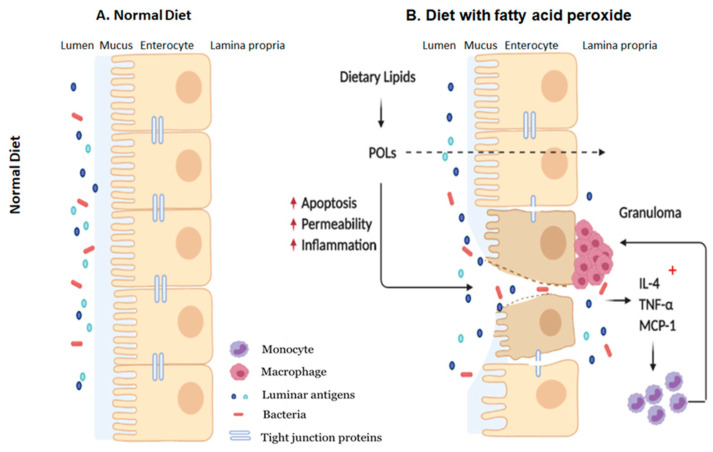
Schematic representation of how dietary POLs interact with the intestinal mucosa. Dietary lipids could be oxidized during normal GI transit to the small and large intestines. Dietary peroxide (13-HPODE) either directly interact with IE cells or diffuse passively. Dietary POLs induce an inflammatory response in the intestine by increasing the expression of pro-inflammatory cytokines IL-4, TNF-α, and MCP-1, that lead to apoptosis, activate immune cells and enhance in monocyte/macrophage recruitment. Apoptosis increases intestinal permeability, which enables luminal toxins/antigens to enter the lamina propria, further inducing inflammation by resident immune cells. TNF-α, Tumor necrosis factor alpha; MCP-1, Monocyte chemoattractant protein-1; IL, Interleukin; POL: Peroxidized lipids.

The findings of this study suggest that POLs may be partly responsible for facilitating inflammation flare-ups in patients with IBD which typically occurs when consuming HFDs, and further validates beneficial nature of restricting dietary fat consumption in these patients. In addition, our results further confirm the immunogenicity of HFDs on the intestinal mucosa and detrimental impact on the gut barrier with effects seen both in the epithelium and resident immune cells (Figure 7). Collectively, our study reaffirms the detrimental impacts of consuming diets high in fats, showing that the effects may manifest as intestinal inflammation from POLs which, in the long run, may be a risk factor for developing and exacerbating IBD.

## Figures and Tables

**Table 1:** Cytokine array: Comparison of changes in inflammatory cytokines in mouse plasma between control and 13-HPODE-fed mice across both acute and chronic time points. Three samples from each group were analyzed by Ray-Bio^®®^ cytokine array analysis. Protein levels are expressed as fold-change between control and peroxide-fed groups. Significance was considered as * *p* < 0.05.

Acute Time-Point				Chronic Time-Point			
Cytokines Upregulated	Fold Difference		*p* Value	Cytokines Upregulated	Fold Difference		*p* Value
	Control	HPODE			NC	NCP	
GCSF	1	2.44717	0.257	GCSF	1	1.883925	0.03639 *
TIMP-1	1	2.30244	0.047 *	TIMP-1	1	1.829084	0.00527 *
sTNF RI	1	2.38687	0.005 *	sTNF RI	1	1.565252	0.21999
BLC	1	1.64366	0.038 *	BLC	1	1.771475	0.03508 *
RANTES	1	2.27539	0.373	RANTES	1	1.61932	0.24741
				MCSF	1	1.648015	0.39222
Fas ligand	1	1.77284	0.193	Eotaxin	1	1.610999	0.1289
KC	1	1.25218	0.600	LIX	1	1.409195	0.11947
Fractalkine	1	1.1411	0.603	IL4	1	1.220213	0.3233
IL-1 beta	1	2.56337	0.405	IL-12p40/p70	1	1.115297	0.32196
				sTNF RII	1	1.105955	0.39748

BLC, B-lymphocyte chemoattractant; GCSF, Granulocyte colony stimulating factor; IL, Interleukin; KC, Keratinocytes-derived chemokine; LIX, C-X-C motif chemokine 5; MCSF, Macrophage colony stimulating factor; RANTES, Regulated upon Activation, Normal T Cell Expressed and Presumably Secreted; sTNFRI, Soluble tumor necrosis factor receptor 1; TIMP-1, Tissue inhibitor of metalloproteinases 1.

## Supplementary Materials

The following are available online at https://www.mdpi.com/2076-3921/9/10/926/s1, Table S1: List of oligonucleotides used for the study; Figure S1: 13-HPODE itself and associated Caco-2 inflammation induces THP-1 chemotaxis.
