# Supplementary material for: Are Fried Foods Unhealthy? The Dietary Peroxidized Fatty Acid, 13-HPODE, Induces Intestinal Inflammation In Vitro and In Vivo
Source: Antioxidants (Basel). 2020 Sep 27;9(10):926. doi: 10.3390/antiox9100926 (PMC7601460; doi:10.3390/antiox9100926)
Supplement: Supplementary file 1 [file antioxidants-09-00926-s001.pdf]

Suplimentary Figure.1: 13-HPODE itself and associated Caco-2 inflammation induces THP-1 chemotaxis

Chemotaxis of THP-1 monocytes was assessed with (A) 13-HPODE alone in the bottom chamber or (B) conditioned media from Caco-2 cells treated for 24 hrs. Results are represented as mean± SEM and significance was considered as *p < 0.05.

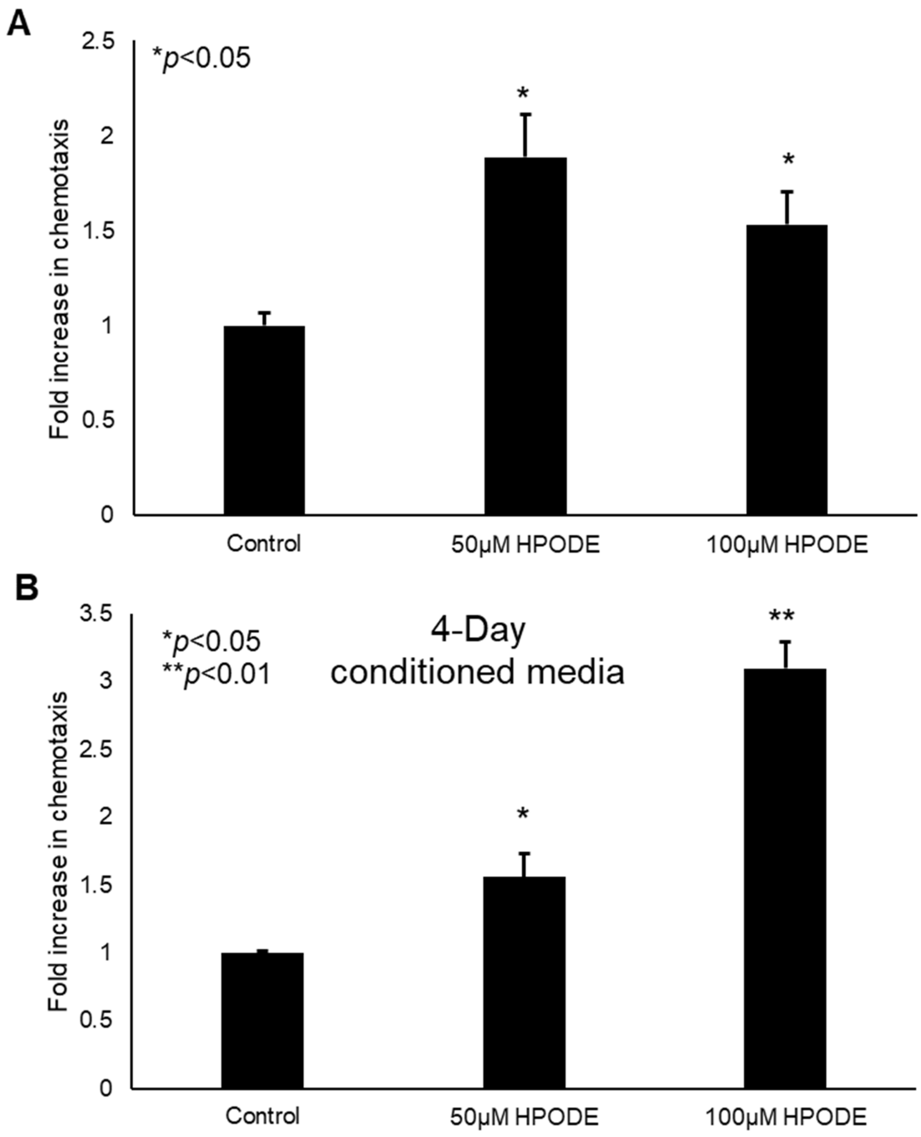

Supplementary Table 1

List of oligonucleotides used for the study

Human	Gene Accession Nos	Forward	Reverse
ALPI	AB011406.1	5'-TCACTCACTCCAAGACCACCA-3'	5'-AAGACTCAGCTCTGTCCTCGGAA-3'
Claudin2	NM_020384.3	5'-CCTTTATCACCTCAGCCCGT-3'	5'-AGGGACTGCTCCCTTGTCTT-3'
GAPDH	NM_001256799.2	5'-AGTCAACGGATTTGGTCGTA-3'	5'-GGAACATGTAAACCATGTAGTTGAG-3'
IL-4	M13982.1	5'-GCACCGAGTTGACCGTAACA-3'	5'-CCAACGTACTCTGGTTGGCT-3'
MCP-1	NM_002982	5'-TCCCCAGACACCCTGTTTTA-3'	5'-GCAATTTCCCAAGTCTCTG-3'
Occludin	U49184.1	5'-ATGGCTGTGCTCTGTGATTACA-3'	5'-GCCAGTTGTGTAGTCTGTCTCA-3'
TNF-α	NM_000594	5'- ATGAGCACTGAAAGCATGATCC-3'	5'-GAGGCTGATTAGAGAGAGGTC-3'

Mouse	Gene Accession Nos	Forward	Reverse
ABCA1	AF287263.1	5'-GGGAATTGAACCTGAGTCCT-3'	5'-AGTCATTCCTCCTCCCATTC-3'
ABCG1	AF323659.1	5'-CCAGACAGTTGTGGATGTGG-3'	5'-GACCTCGCTCTTCCTTCCTT-3'
ALPI	AB473959.1	5'-GTACCCGAAGAACAGAACCGAC-3'	5'-TAGTCCACCCTGGAGGGGTC-3'
CD36	NM_001159555	5'-TGCTGGAGCTGTTATTGGTG-3'	5'-TGGGTTTTGCACATCAAAGA-3'
Claudin2	NM_016675.4	5'-ATGCCTTCTTGAGCCTGCTT-3'	5'-AAGGCCTAGGATGTAGCCCA-3'
GAPDH	GU214026	5'-ACCCAGAAGACTGTGGATGG-3'	5'-CACATTGGGGGTAGGAACAC-3'
IL-4	NM_021283.2	5'-GCGACAAAAATCACTTGAGAG-3'	5'-CCTTGGAAGCCCTACAGAC-3'
IL-6	NM_031168.2	5'-AGTTGCCTTCTTGGGACTGA-3'	5' -TCCACGATTTCCCAGAGAAC-3'
MCP-1	NM_011333.3	5'-CAGCAAGATGATCCCAATGA-3'	5'-TGGTTCCGATCCAGGTTTT-3'
Occludin	U49185.1	5'-CCCTCTTTCCTTAGGCGACA-3'	5'-GAGTACGCTGGCTGAGAGAG-3'
SRA1	NM_001113326.1	5'-AAAGGTGATCGGGGACAAA-3'	5'-TTGCCCCAATATGATCAGG-3'
SRB1	NM_016741.2	5'-GGGCTCGATATTGATGGAGA-3'	5'-GGAAGCATGTCTGGGAGGTA-3'
TNF-α	NM_001278601.1	5'-CACACTCAGATCATCTTCCAAAA-3'	5'-GCAATGACTCTAAGTAGACCTGC-3'
